# Public health round-up

**DOI:** 10.2471/BLT.25.010725

**Published:** 2025-07-01

**Disability Health Equity Initiative**

The World Health Organization (WHO) has launched the WHO Disability Health Equity Initiative, a landmark global initiative to advance health equity for over 1.3 billion people with disabilities. Unveiled on 10 June 2025, at the United Nations Headquarters in New York during the 18th session of the Conference of States Parties to the Convention on the Rights of Persons with Disabilities, the initiative aims to guide governments, health institutions, and communities in addressing barriers to care, promoting inclusive policies, and strengthening data and research on disability and health.

**Figure:**
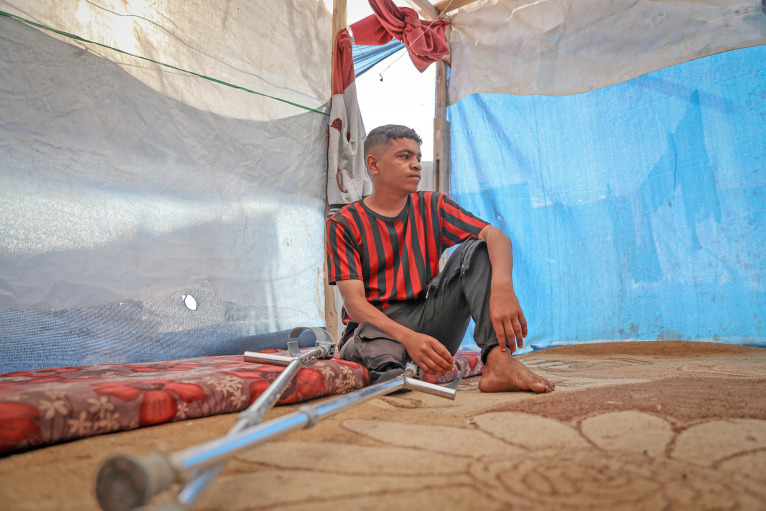
WHO

## Tobacco control progress report

The World Health Organization (WHO) released its *Global Tobacco Epidemic 2025* report at the World Conference on Tobacco Control in Dublin, Ireland, urging governments to strengthen tobacco control policies amid rising industry interference. Despite notable progress (with over 6.1 billion people now protected by at least one WHO MPOWER measure) serious gaps remain.

Only four countries (Brazil, Mauritius, the Kingdom of the Netherlands and Türkiye) have fully implemented the MPOWER package, while 40 countries still lack even a single measure at best-practice level. Although 110 countries now require graphic health warnings on tobacco packaging, enforcement and comprehensive campaigns remain uneven. Quit services, taxation and bans on advertising also lag in many regions.

WHO Director-General Tedros Adhanom Ghebreyesus called for renewed urgency: “By uniting science, policy and political will, we can create a world where tobacco no longer claims lives, damages economies or steals futures.”

The report highlights a growing trend to regulate e-cigarettes, however over 60 countries still lack any electronic nicotine delivery systems oversight. WHO calls for urgent, coordinated global action to close the remaining gaps.

https://bit.ly/446nXwY

## Origins of COVID-19

The World Health Organization’s Scientific Advisory Group for the Origins of Novel Pathogens (SAGO) has released its report evaluating the origins of SARS-CoV-2. The 27-member international panel reviewed scientific literature, fieldwork and unpublished data over three years. While the report highlights progress in understanding how the coronavirus disease 2019 (COVID-19) emerged, it stresses that key information, particularly from China, remains unavailable.

The report concludes that the weight of available evidence suggests zoonotic spillover, possibly from bats directly or through an intermediate host. However, it acknowledges that other hypotheses, including a laboratory-related incident, cannot yet be ruled out due to significant gaps in the information available to evaluate the various hypotheses. 

WHO Director-General Tedros Adhanom Ghebreyesus reiterated the need for full transparency: “As things stand, all hypotheses must remain on the table, including zoonotic spillover and lab leak. We continue to appeal to China and any other country that has information about the origins of COVID-19 to share that information openly.”

SAGO Chair Dr Marietjie Venter emphasized the broader significance of the work: “This is not solely a scientific endeavour, it is a moral and ethical imperative to help prevent future pandemics.”

https://bit.ly/4lrwEYl

## Demand for release of staff detained in Yemen 

This June marks one year since dozens of staff from the United Nations (UN), international nongovernmental organizations (INGO), civil society organizations and diplomatic missions were arbitrarily detained by Houthi authorities in northern Yemen. In a joint petition, the heads of UN agencies and INGOs urgently renew their call for the immediate and unconditional release of all those detained. 

Currently, 23 UN and five INGO staff remain in detention. Tragically, one UN staff member and a Save the Children employee have died while detained. These detentions violate international law and cause immense suffering for both detainees and their families.

While a few individuals have been released, signatories of the petition urge Houthi authorities to fulfill past commitments and release all remaining detainees. 

https://bit.ly/3GphhAX

## Midwifery models of care

WHO has released new global guidance advocating for the expansion of midwifery-led models of care, calling them a vital, cost-effective strategy to improve outcomes for mothers and babies. *Implementation guidance on transitioning to midwifery models of care* explains that empowering midwives to serve as primary care providers throughout pregnancy, childbirth and the postnatal period leads to better health outcomes, fewer unnecessary medical interventions, and greater patient satisfaction.

“Expanding and investing in midwifery models of care is one of the most effective strategies to improve maternal and newborn health globally,” said Dr Anshu Banerjee, WHO director for Maternal, Newborn, Child and Adolescent Health and Ageing.

Universal access to skilled midwives could prevent over 60% of maternal and newborn deaths, saving an estimated 4.3 million lives annually by 2035. The guidance highlights tools for implementation, stresses the importance of regulation and education, and outlines adaptable models such as continuity of care and midwife-led birth centres.

WHO and partners urge governments to prioritize political will, financing, and system integration to ensure respectful, person-centered care for every woman and child.

https://bit.ly/3I94oeN

## Energy progress report

*Tracking SDG 7: the energy progress report 2025* released on 25 June 2025 states that while 92% of the global population now has basic access to electricity, over 670 million people remain without access, and over 2 billion people remain dependent on polluting and hazardous fuels such as firewood and charcoal for their cooking needs. 

The report emphasizes that at the current pace, universal access to electricity and clean cooking by 2030 remains out of reach.

Despite international financial flows for clean energy in developing countries reaching 21.6 billion United States dollars in 2023, regional disparities persist due to financing and infrastructure gaps.

The report highlights the role of decentralized renewable energy, such as mini-grids and off-grid solar, as key to reaching remote and underserved communities. The report also emphasizes the urgent need for sustained international financial support to enhance energy infrastructure and ensure equitable access. It calls on governments, international organizations and the private sector to collaborate in mobilizing funds and implementing policies that prioritize energy access as a fundamental right.

“We must accelerate progress at this crunch time,” urged Francesco La Camera, Director General of the International Renewable Energy Agency. “To close the access and infrastructure gaps, we need strengthened international cooperation to scale up affordable financing and impact-driven capital for the least developed and developing countries.”

https://bit.ly/3I6xUBU

## Protection of hospitals in the Gaza Strip

WHO has issued an urgent appeal to safeguard Nasser Medical Complex and Al-Amal Hospital in Khan Younis, Gaza Strip. These two hospitals are the last functioning public facilities in southern Gaza, where most of the population now resides. Northern Gaza currently has no operational hospitals.

Both hospitals are overwhelmed and operating far beyond capacity, while struggling with severe shortages of medicines and supplies. Israeli authorities have indicated access routes to the hospitals will be obstructed, threatening their ability to remain functional. 

Losing the two hospitals would cut 490 beds, reducing the Gaza Strip’s overall hospital bed availability to less than 1400 hospital beds (40% less hospital beds available than before the start of the conflict), for an entire population of 2 million people.

 WHO calls for the protection of medical facilities, safe humanitarian access and an immediate ceasefire.

https://bit.ly/3I3kTZT

## Pregnancy and sickle cell disease

WHO has released its first global guideline on managing sickle cell disease (SCD) during pregnancy, addressing a growing health challenge with life-threatening risks for both women and babies. SCD, characterized by abnormal crescent-shaped red blood cells, can lead to severe complications, including strokes, infections, and organ failure. Pregnant women with SCD face a significantly higher risk of maternal death and obstetric complications.

“Quality health care can ensure safe pregnancies for women with inherited blood disorders like sickle cell disease,” said Dr Pascale Allotey, WHO’s director for Sexual and Reproductive Health and Research. The guideline provides over 20 evidence-based recommendations covering pain relief, infection prevention and blood transfusions, with a focus on respectful, individualized care. It emphasizes the need for skilled care teams and highlights the urgency of research into safe treatment options for pregnant women with SCD.

The guideline aims to improve outcomes in low- and middle-income countries, where most SCD cases and deaths occur.

https://bit.ly/4l5UTeZ

**Cover photo**

A fisherman in Palau. In June 2025, during the United Nations Ocean Conference in Nice, France, 19 countries ratified the High Seas Treaty. With these new endorsements, the total number of ratifications has reached 50, just ten away from the required for the treaty to enter into force.

**Figure:**
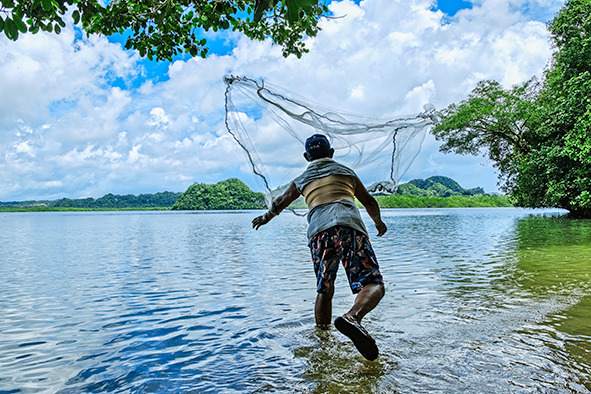
(WHO/Yoshi Shimizu)

**Looking ahead**

11 July. High-level Interactive Dialogue on the Social, Economic and Environmental Determinants of Health, UN Headquarters, New York. https://bit.ly/3ZPxO7W

14–18 July. 2nd Global Convening of the Global Initiative on Digital Health, virtual event. https://bit.ly/40poDLw

25 July. World Drowning Prevention Day, global events. https://bit.ly/3ZSfKtL

28 July. World Hepatitis Day 2025: Let’s Break It Down, global events. https://bit.ly/4lArEky

